# Camptothecin-20(s)-*O*-[*N*-(3’α,12’α-dihydroxy-24’-carbonyl-5’β-cholan)]-lysine, a Novel Camptothecin Analogue, Induces Apoptosis towards Hepatocellular Carcinoma SMMC-7721 Cells

**DOI:** 10.3390/molecules16097803

**Published:** 2011-09-13

**Authors:** Qingyong Li, Wei Qiu, Qiaochu Zhu, Yuangang Zu, Xiaoqiu Deng, Tengfei Zhao, Chunfei Jiang, Li Zhang

**Affiliations:** The Key Laboratory of Forest Plant Ecology, Northeast Forestry University, Ministry of Education, Harbin 150040, China; E-Mails: qiuwei3042@126.com (W.Q.); zhuqiaochu1987@yahoo.com.cn (Q.Z.); zygorl@vip.hl.cn (Y.Z.); 103077522@qq.com (X.D.); zz0111@hotmail.com (T.Z.); jiangchunfei2009@126.com (C.J.); ally521@126.com (L.Z.)

**Keywords:** camptothecin analogue, apoptosis, hepatocellular carcinoma SMMC-7721 cell

## Abstract

Camptothecin-20(s)-*O*-[*N*-(3’α,12’α-dihydroxy-24’-carbonyl-5’β-cholan)]-lysine (B2) is a novel camptothecin analogue. Our previous study had shown that it displayed higher cytoxicity activity towards hepatocellular carcinoma SMMC-7721 cells than camptothecin (CPT) *in vitro*. In this paper, the underlying mechanism of anti-proliferation of B2 towards SMMC-7721 cells was further examined. Cell growth inhibition of B2 was determined using the 3-(4,5-dimethylthiazol-2-yl)-2,5-diphenyltetrazolium bromide (MTT) assay; morphological changes were observed under Laser Scanning Confocal Microscope (LSCM); cell cycle distribution, apoptotic population, changes in mitochondrial membrane potential, intracellular calcium concentration and reactive oxygen species (ROS) production were determined by flow cytometry (FCM). Activities of caspase-3 and caspase-9 were measured, and the expression level of Bcl-2 and Bax proteins were analyzed by Western blot. The results suggested that B2 inhibited SMMC-7721 cell growth by causing cell cycle arrest at the S and G2/M phases, and induced apoptosis involving a mitochondrial pathway. B2 appears to cause a high induction of apoptosis on SMMC-7721 cells *in vitro*, which suggests it might be a potential drug for cancer therapy.

## 1. Introduction

Hepatocelluar carcinoma (HCC) is one of the most common malignancies in the World. According to the World Health Organization, 42% of HCC cases occur in China each year [1]. It caused by unlimited proliferation and migration of cancer cells, and frequently is diagnosed as one of common solid tumors in the liver [2]. Therefore, the detection of HCC is difficult and majority of the patients are diagnosed at an advanced stage of the disease. It is unfortunate that HCC is very refractory to traditional chemotherapy, radiation therapy, and even immunotherapy [3]. Thus, new anti-tumor drugs need to be designed and investigated.

**Figure 1:**
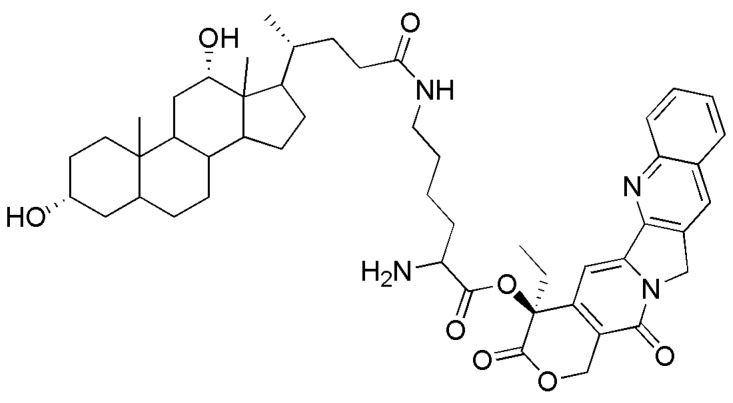
The structure of B2.

Camptothecin (CPT) is an alkaloid originally isolated from *Camptotheca acuminate* by Wall and Wani in 1966 [4]. CPT and its natural synthetic analogs showed high anti-tumor activity *in vitro* and *in vivo*, however CPT’s toxicity towards normal tissues led to the termination of its clinical trials [5]. Among the developments in the field of chemotherapeutic agents, anti-tumor targeting drugs are attracting great attention. In previous studies, a series of novel camptothecin-bile acid analogues were synthesized to improve the targeting activity and reduce the toxicity of CPT. The bile acids were used as shuttles for directing CPT to the liver via the entero-hepatic circulation. The anti-tumor activities of these analogues were evaluated towards to HCT-116 cells, SMMC-7721 cells, BEL-7402 cells and Hela cells. Some of the analogues showed stronger cytotoxicity to human liver cancer cells and low toxicity to normal liver cell compared to that of CPT. Among these derivatives, camptothecin-20(s)*-O-*[*N*-(3’α,12’α-dihydroxy-24’-carbonyl-5’β-cholan)]-lysine (B2, Figure 1) demonstrated super cytotoxicity towards the human hepatocarcinoma cancer SMMC-7721 cell line than CPT, and competition assays indicated that bile acids played an important role to carry B2 into the bile acid–positive cancer cells. The cytotoxic activities of B2 against bile acid–negative Hela cells were much lower. The topoisomerase I inhibition of B2 was 1.75 times that of CPT [6]. However, the mechanisms of B2 against human hepatocarcinoma cancer cells remained unknown. Therefore, the present study was carried out to evaluate the effects of B2 on human hepatocarcinoma cancer cells proliferative using MTT assay, cell cycle distribution, the various factors of apoptosis, Bcl-2 and Bax protein expression.

## 2. Results and Discussion

### 2.1. Anti-Proliferative Activity of B2

**Figure 2:**
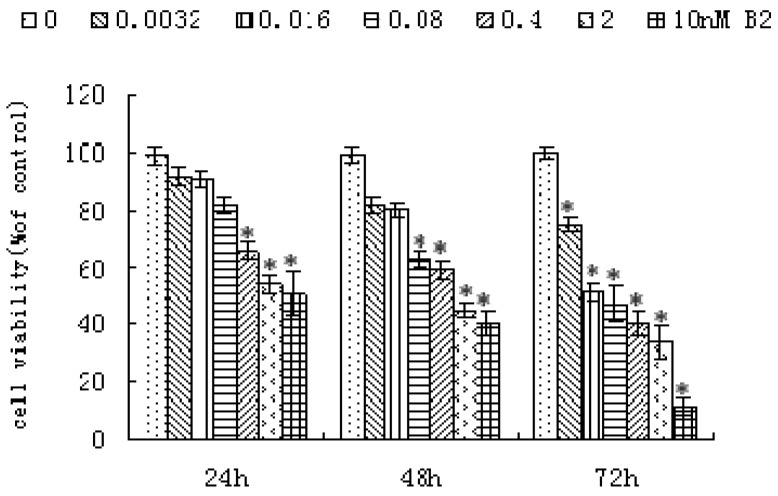
Effects of B2 on the growth of SMMC-7721 cells. SMMC-7721 cells were treated with B2 for 24, 48 and 72 h at the indicated concentrations, and cells viability was measured by an MTT assay. The data was expressed as means ± SD of three separate experiments. * *p* < 0.05, *p* value compared with the control group (0 nM).

The anti-proliferative activity of B2 was determined in SMMC-7721 cells by the MTT assay. As shown in Figure 2, the growth of SMMC-7721 cells was significantly inhibited in a dose- and time- dependent manner by increasing concentrations of B2 after 24, 48 and 72 h. When SMMC-7721 cells were treated with 0.4 nM B2, 41.26% of cells were killed after 48 h.

**Figure 3:**
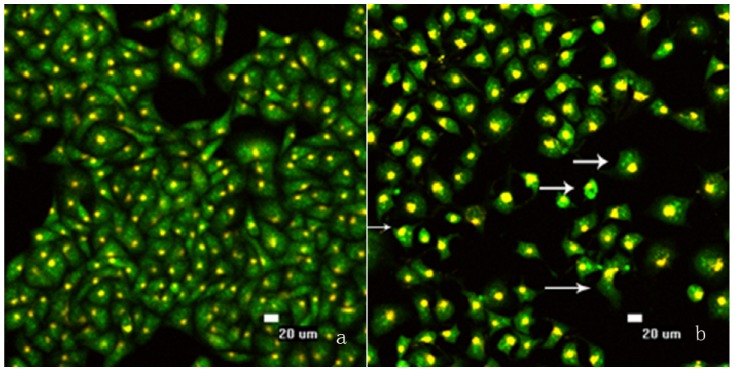
Morphological changes in SMMC-7721 cells treated with B2 for 48 h examined by LSCM. The morphological changes of cells are indicated by arrows. (**a**) Control cells; (**b**) Cells treated with 0.5 nM B2.

In order to further explore the effects of B2 treatment on the cells, morphological observation was performed. Both the control cells and cells treated with 0.5 nM B2 for 48 h were viewed under the LSCM (Figure 3). 

A shown in Figure 3, the control cell membranes were orbicular and the nuclei were sharp and regular, but cells treated with B2 displayed typical apoptotic features such as nuclear and cytoplasmic condensation, chromatin fragmentation, marginalization of the fragmented nuclei towards the membrane, and apoptotic bodies were formed. All of these changes suggested that B2 had significant cytotoxicity toward SMMC-7721 cells.

### 2.2. FCM Analysis of SMMC-7721 Cell Cycle Distribution and Apoptosis

**Figure 4:**
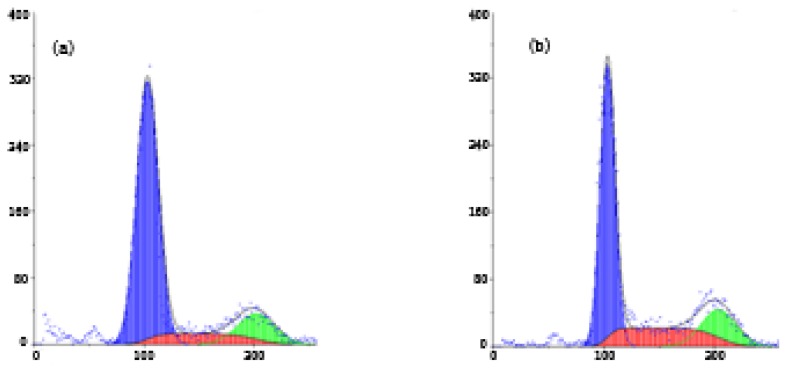

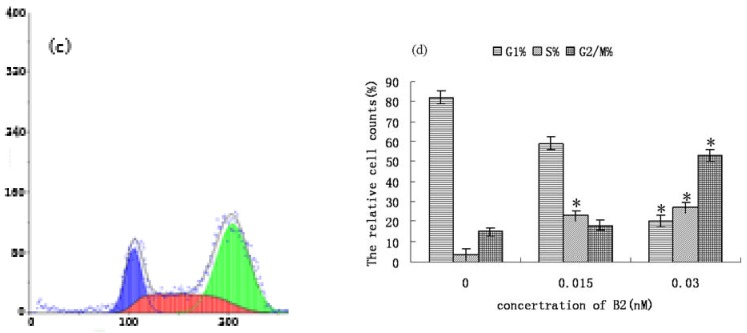
Flow cytometry analyses of nuclear DNA content. SMMC-7721 cells were treated with 0 nM B2 (**a**); 0.015 nM B2 (**b**) and 0.03 nM B2 (**c**) for 48 h. The *x*-axis represents fluorescence intensity on a logarithmic scale, and the *y*-axis represents the number of events. The results in (**d**) were analyzed by Mod Fit LT 3.0. Each experiment was performed in triplicate. * *p* < 0.05, *p* value compared with the control group (0 nM).

Apoptosis is a physiological process that functions as an essential mechanism of tissue homeostasis and is regarded as the preferred way to eliminate unwanted cells [7]. CPT and its derivatives are able to form cleavable drug-enzyme-DNA complexes that inhibit the DNA relegation step [8]. Collision between the DNA replication fork and these complexes has been proposed as a means of explaining the CPT-driven S phase-specific cytotoxicity and the arrest of cells in the G2/M phase of the cell cycle [9]. As shown in Figure 4, a significant increase in the percentage of cells in S phase was found after treatment with B2, compared with control cells (from 3.66% to 26.94%). Meanwhile, a significant increase in G2/M phase was also found (from 14.75% to 53.19%). The result suggested that the anti-proliferative effect of B2 may be related to the accumulation of cells in the S and G2/M phases. Furthermore, we considered the camptothecin-bile acid analogue retained the characteristics of CPT and arrested the cell cycle. To further confirm that the inhibition of cell growth by B2 is related to apoptosis, Annexin V-FITC and PI double staining-based flow cytometry analysis was performed on SMMC-7721 cells. Annexin-V binding is based on the transposition of phosphatidylserine from the inner to the outer face of the cell membrane during the early stages of apoptosis [10].

PI was used to differentiate apoptotic cells with membrane integrity (Annexin V+/PI−) from that had lost membrane integrity (Annexin V+/PI+) [11]. The fraction of the cell population in different quadrants was analyzed using quadrant statistics. 

**Figure 5:**
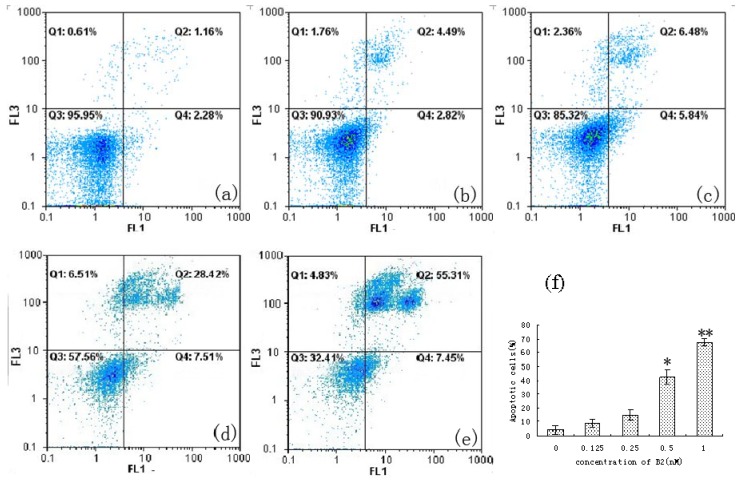
Apoptotic population of SMMC-7721 cells analysis by flow cytometric using annexin V-FITC/PI staining. Q1, necrotic cells (annexin V−/PI+); Q2, late apoptotic cells (annexin V+/PI+); Q3, viable cells (annexin V−/PI−); Q4, early apoptotic cells (annexin V+/PI−). (**a**) 0 nM; (**b**) 0.125 nM; (**c**) 0.25 nM; (**d**) 0.5 nM; (**e**) 1 nM B2 for 48 h; (**f**), the percentage of annexin V positive population on every concentration of B2. ** *p* < 0.01, * *p* < 0.05, *p* value compared with the control group (0 nM).

The annexin-V positive and PI negative cells were early-stage apoptotic cells, whereas annexin-V positive and PI positive cells were late stage apoptotic cells. As shown in Figure 5, the results showed that B2 could induce dose-dependent apoptosis in SMMC-7721 cells. The percentage of late stage apoptotic cells was increased from 1.16% to 55.31%. The percentage of apoptotic cells changed significantly when the concentration of B2 reached 0.5 nM. Taken together, we conclude that B2 inhibited SMMC-7721 cells growth through induction of apoptosis.

### 2.3. B2 Induces SMMC-7721 Apoptosis via Mitochondrion Pathway

The mechanism of camptothecin-induced apoptosis is definite, and the mitochondrial apoptotic pathway is one major pathway of camptothecin-induced apoptosis [12,13]. Mitochondria play a central role in the regulation of apoptotic signaling [14,15]. To evaluate the role of mitochondria in B2-induced apoptosis, we carried out a series of experiments. Ca^2+^ is an important signal transducer, not only for intracellular communication but also for intercellular communication. Many studies have demonstrated that an increase in intracellular Ca^2+^ plays a primary role in triggering the apoptosis pathway [16]. The increase in intracellular Ca^2+^ can induce a loss of mitochondrial membrane potential (Δ*Ψ* m) [17]. Disruption of Δ*Ψ* m is one of the earliest intracellular events in apoptosis induction [13,18], and this reduction in Δ*Ψ* m is often accompanied by the production of reactive oxygen species ROS [19]. Caspases, the cytoplasmic aspartate-specific cysteine proteases, also play an important role in apoptosis [20]. The mitochondrion-dependent pathway requires activation of caspase-9. The increase of ROS can induce activation of caspase-9 [21]. Subsequently, caspase-9 can activate caspase-3, which in turn targets and degrades specific and vital cellular proteins, ultimately resulting in nuclear DNA degradation and apoptotic death of the cells [22].

**Figure 6:**
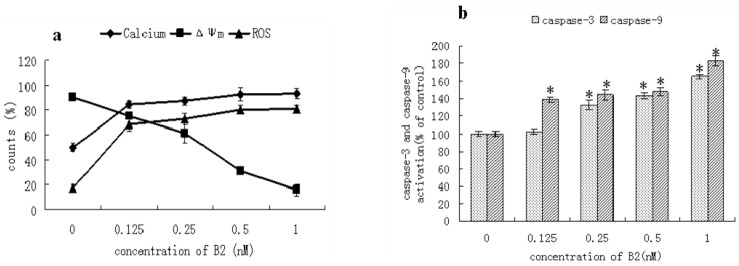
(**a**) The changes of calcium, mitochondrial membrane potential (Δ*Ψ* m) and ROS were analysis by FCM. SMMC-7721 cells were treated with the desired concentration of B2 for 48 h. The data represent the percentages of cells within the specified fluorescence intensity range using WinMDT 2.9 software; (**b**) Effect of B2 on caspase-3 and caspase-9 activities. The activity was represented the absorbance of cells treated with B2 compared with the absorbance of control cells. The values were representative of three separate experiments. *p < 0.05, *p* value compared with the control group (0 nM).

As shown in Figure 6(a), the treatment of SMMC-7721 cells with different concentrations of B2 resulted in an increase of Ca^2+^ from 79.44% to 93.46%. Furthermore, the Δ*Ψ* m was decreased from 77.94% to 15.74%, and this change induced an obvious increase of ROS from 29.71% to 80.81%. As shown in Figure 6(b), the activation of caspase-3 or caspase-9 was dose-dependent. The activation of caspase-3 or caspase-9 showed an obvious increase when the concentration of B2 reached 0.25 nM or 0.125 nM, respectively. Therefore, B2 could induce an increase of the intracellular Ca^2+^ concentration, and caused the loss of Δ*Ψ* m followed an increased production of ROS. The changes of ROS induced by B2 activated caspase-9, which could activate caspase-3. These changes are characteristics of a mitochondrion-dependent apoptotic pathway. We initially concluded that B2 induced SMMC-7721 cells apoptosis through the mitochondrion apoptotic pathway.

### 2.4. Apoptosis-Related Proteins in SMMC-7721 Treated with B2

**Figure 7:**
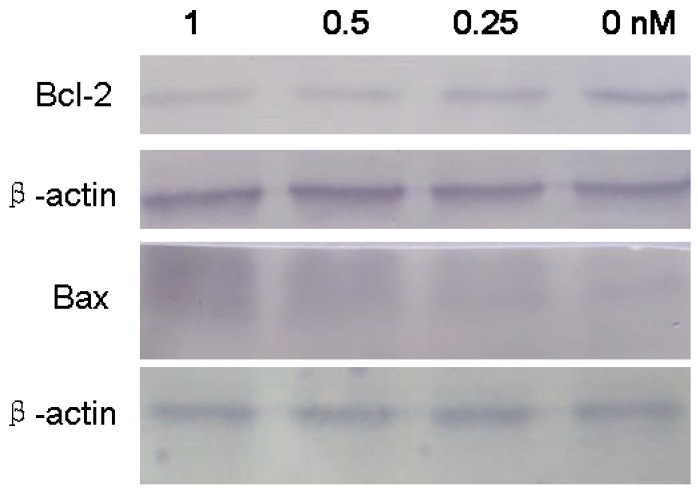
Effect of B2 on the expression of Bcl-2 and Bax. SMMC-7721 cells were treated with desired concentration of B2 for 48 h. Anti-apoptotic Bcl-2 and pro-apoptotic Bax were analyzed by western blot; β-actin was used as a control for loading.

The Bcl-2 family proteins have a central role in controlling the mitochondrial pathway. The pro-apoptotic proteins and anti-apoptotic proteins of the Bcl-2 family can form heterodimers, which may turn apoptosis on and off [23,24]. Bcl-2 is an anti-apoptotic protein, and Bax is a pro-apoptotic protein. In our research, B2 induced SMMC-7721 apoptotic, which was accompanied by down-regulation of Bcl-2 and up-regulation of Bax (Figure 7). It has been reported that Bcl-2 suppressed ROS-induced apoptosis and the overexpression of the Bax enhanced ROS generation. The increase of Bax/Bcl-2 rations may support the theory that B2-induced apoptosis is mediated through a mitochondrial oxygen stress pathway.

## 3. Experimental

### 3.1. Materials

RPMI-1640 medium was purchased from HyClone (Logan, UT, USA). Fetal bovine serum (FBS), penicillin, streptomycin and trypsinase were purchased from Hao Yang Biological Manufacture Co., Ltd (Tianjin, China). MTT, acridine orange (AO), dimethyl sulfoxide (DMSO), Fluo-3/AM and rhodamine 123 (Rh123) were purchased from Sigma Chemical Co (St. Louis, MO, USA). Phosphate-buffered saline (PBS, PH 7.2–7.6) was purchased from Wuhan Boster Biological Technology Ltd (Wuhan, China). Cystain was purchased from Partec GmbH (Münster, Germany). Annexin V-FITC apoptosis detection kit, Caspase-3 Colorimetric Assay Kit and Caspase-9 Colorimetric Assay Kit were purchased from KeyGen Biotech Co (Nanjing, China). Reactive Oxygen Species Assay (ROS) Kit, Western Blotting Kit, BCA Protein Assay Kit, BCIP/NBT Alkaline Phosphatase Color Development Kit and Anti-Bcl-2 antibody were purchased from the Beyotime Institute of Biotechnology (Heilongjiang, China). Anti-Bax antibody was purchased from Santa Cruz Biotechnology (Delaware Avenue Santa Cruz, CA, USA). B2 was synthesized as described in our precious report. The B2 stock solution was prepared in DMSO and stored at 4 °C in the dark. Dilutions of the compounds were made up in 2% culture medium before each experiment. The final concentration of DMSO did not exceed 0.1% (v/v) and was nontoxic to the cells.

### 3.2. Cell Culture

Hepatocellular carcinoma SMMC-7721 cells were obtained from the Cell Bank of Shanghai Institute of Biochemistry and Cell Biology, Chinese Academy of Sciences (Shanghai, China). The cells were cultured in RPMI-1640 medium supplemented with 10% FBS and 100 U/mL penicillin-100 mg/L streptomycin in a humidified atmosphere of 5% CO_2_ at 37 °C.

### 3.3. Cell Proliferation Assay

Cell proliferation was measured using an MTT assay [25]. SMMC-7721 cells were plated in 96-well plates at a density of approximately 85%. After treatment with B2 (0.0032–10 nM) for 24, 48 and 72 h, MTT solution (5 mg/mL in phosphate-buffered saline) was added (20 µL/well), and the plates were incubated for another 4 h at 37 °C. All media was removed and the formazan crystals were dissolved in 100 µL DMSO per well. Absorbance was read at 570 nm (630 nm as a reference) on a microplate reader (Awareness Technology, Palm City, USA). Assays were performed in three independent experiments.

### 3.4. Morphological Observation of Nuclear Change

Following by treated with B2 for 48 h, SMMC-7721 cells were washed and stained with AO (200 µL, 20 µg/mL). After incubating 10 min in dark, the plate was washed with cold PBS twice, and viewed under the Laser Scanning Confocal Microscope (Nikon Eclipse TE2000-E, Tokyo, Japan) [26,27].

### 3.5. Apoptosis Assays and Cell Cycle Analysis

Briefly, SMMC-7721 cells were seeded in a 6-well plate for 24 h and exposed to B2 for 48 h. Then the cells were harvested and washed with PBS. The cell pellets were re-suspended in PBS (800 µL) and stained with CyStain (200 µL) for 10 min in the dark [28]. The cell cycle was determined by flow cytometry (Partec GmbH, Germany). Apoptosis assays were performed using an annexin V-FITC apoptosis detection kit following the manufacturer’s instructions. After exposure to B2 for 48 h, the cells were collected and washed with PBS twice, gently re-suspended in annexin V-FITC binding buffer (195 µL) and incubated with annexin V-FITC (5 µL) for 10 min at room temperature in the dark. The cells were then centrifuged at 3,000 rpm for 5 min, gently re-suspended in annexin V-FITC binding buffer (190 µL) and PI (10 µL) was added, followed by immediate analysis by flow cytometry [29,30].

### 3.6. Assessment of Mitochondrial Membrane Potential (ΔΨ m)

Δ*Ψ* m was measured by flow cytometry with Rh123 [31,32]. SMMC-7721 cells were plated in a 6-well plate, and exposed to different concentrations of B2 for 48 h. Then cells were washed with PBS and finally harvested in PBS (1 mL) containing Rh123 (10 µg/mL). The samples were incubated for 30 min at 37 °C in dark. The cells were washed and analyzed immediately by flow cytometry at 488 nm.

### 3.7. Determination of the Changes of Calcium and ROS in Cells

SMMC-7721 cells were seeded in a 6-well plate. After treatment with the desired concentration of B2 for 48 h, the cells were harvested and washed with PBS, and then Fluo-3/AM (10 µL) was added for 30 min at 37 °C in the dark [33]. The samples were re-suspended in PBS and analyzed immediately by flow cytomertry.

The extent of ROS production was measured through a Reactive Oxygen Species Assay Kit [3]. After treated with B2 for 48 h, the cells washed with PBS and incubated with 10 µM dihydrodichloro-fluorescein diacetate (DCFH-DA). Intracellular DCFH-DA was deesterfied to dichlorodihydro-fluorescein (DCFH) which is oxidized by ROS to produce the fluorescent compound DCFH. After incubation for 20 min at 37 °C in the dark, the cells were analyzed for fluorescence intensity by flow cytometry.

### 3.8. Measurement of Caspase-3 and Caspase-9 Activities

The activations of caspase-3 and caspase-9 were determined with a colorimetric assay kit [34]. SMMC-7721 cells were treated with B2 for 48 h, then harvested and washed with PBS. After the cells were lysed, reaction buffer was added to the samples followed by additional caspase-3 or caspase-9 colorimetric substrate (DEVD-pNA, 5 µL) and incubated in a 96-well plate for 6 h at 37 °C in a CO_2_ incubator. The absorbance value at 405 nm was read with a microplate reader.

### 3.9. Western Blot Analysis

After the drug treatment, SMMC-7721 cells were collected and washed with cold PBS, then treated with ice-cold lysis buffer with 1% PMSF (phenylmethylsulfonyl fluoride). Cell lysates were centrifuged at 12,000 × g at 4 °C for 5 min, then the supernatants were collected, and the protein concentration was determined by the BCA Protein Assay Kit. Equal amounts of protein lysates were subjected to electrophoresis on 12% SDS-PAGE and transferred onto PVDF membranes. Non-specific binding was reduced by incubating in blocking buffer for 2 h at room temperature. After blocking, the membrane was incubated with specific primary antibodies (anti-Bcl-2 and anti-Bax antibodies) overnight at 4 °C and further incubated for 1 h with alkaline phosphatase labeled respective secondary antibodies. The membrane was incubated with BCIP/NBT solution until color development was achieved. The reaction was stopped by the addition of distilled water.

### 3.10. Statistical Analysis

Results were expressed as mean ± SD. Statistical analysis was performed using Student’s t-test. Significance was established at *P* <0.05.

## 4. Conclusions

Our study has demonstrated that B2, a camptothecin analog, exhibited anti-proliferative effects on hepatocellular carcinoma SMMC-7721 cells in a dose- and time-dependent manner by induction of apoptosis. B2-induced cell apoptosis associated with cell cycle arrest and the activation of the mitochondrial apoptotic pathway. However, further research is required to establish the targeting activity *in vivo* of B2.
